# Quadriceps neuromuscular function in women with patellofemoral pain: Influences of the type of the task and the level of pain

**DOI:** 10.1371/journal.pone.0205553

**Published:** 2018-10-10

**Authors:** Ronaldo Valdir Briani, Danilo De Oliveira Silva, Carolina Silva Flóride, Fernando Amâncio Aragão, Carlos Eduardo de Albuquerque, Fernando Henrique Magalhães, Fábio Mícolis de Azevedo

**Affiliations:** 1 Physical Therapy Department, School of Science and Technology, São Paulo State University, Presidente Prudente, Sao Paulo, Brazil; 2 Department of Physical Therapy, State University of West of Parana, Research Laboratory of Human Movement, Cascavel, Paraná, Brazil; 3 School of Arts, Sciences, and Humanities, University of São Paulo, São Paulo, Brazil; University of Tennessee Health Science Center College of Graduate Health Sciences, UNITED STATES

## Abstract

The present study aimed at investigating whether the neuromuscular system behaves differently (in terms of force and muscle activity generation) as a function of the task being performed (i.e. maximal voluntary efforts *vs* stair negotiation) and the presence of patellofemoral pain (PFP) and possible influences of pain intensity. Thirty-eight women with (n = 19) and without PFP (n = 19) had their knee strength (extension joint torque) measured during maximal voluntary isometric contractions (MVIC) and electromyography (EMG) data recorded during both MVIC and stair ascent tasks, which were performed before and after a loading protocol designed to exacerbate pain symptoms. Women with PFP displayed lower levels of vastus medialis (p = 0.002) and vastus lateralis (p = 0.032) EMG activation during MVIC assessments. Conversely, the PFP group showed higher levels of vastus medialis muscle activity during stair climbing (p = 0.007), which happened exclusively after the loading protocol. Similarly, women with PFP displayed lower knee extensor torque only during the MVIC tests performed after the loading protocol, which was moderately correlated with the increase in self-reported pain (p = 0.041, r = 0.37), whereas the changes in EMG activity during stair ascent were not correlated with changes in pain intensity (p = 0.215, r = 0.12). These results suggest that, in comparison to pain-free controls, women with PFP display lower levels of quadriceps EMG activation during maximal contractions, but higher activation during dynamic tasks (stair ascent). In addition, the moderate association between the decrease in knee extensor torque and increase in self-reported pain indicates that care should be taken by clinicians during quadriceps strength evaluation in women with PFP, as misleading outcomes may emerge if the intensity of knee pain is not considered during screening. Additionally, rehabilitation strategies should focus on both restoring neuromuscular control and increasing muscle strength.

## Introduction

Patellofemoral pain (PFP) represents a common condition observed in orthopedic practice characterized by diffuse peripatellar and retropatellar pain [1]. PFP has an annual prevalence of 22.7% in the general population [2,3], affecting mainly physically active young women which are 2.23 times more prone to develop PFP as compared to men [4]. PFP is typically exacerbated by activities of daily living that require loading on a flexed knee such as stair walking [5]. In addition, non-weight-bearing quadriceps contractions are also known to increase compressive forces at the patellofemoral joint (PFJ) thereby contributing to PFP symptoms [6].

Several studies have investigated the contribution of altered quadriceps function to the development of PFP [7–11]. Decreased torque, total volume, cross-sectional area [11], altered electromyographic (EMG) activity [7,9,10] and strength [7,8] of the quadriceps muscle have been reported in subjects with PFP as compared to asymptomatic subjects. Given the contribution of quadriceps femoris on controlling patellar movement during knee flexion–extension [12,13], these alterations are thought to contribute to abnormal patellar tracking that causes excessive compression to the lateral patella facets [12–14]. In this line of reasoning, the literature provides evidence for the use of quadriceps-strengthening exercises for the treatment of PFP [15], although better results can be achieved by combining knee and hip exercises [16].

Nevertheless, controversial findings have been reported as to altered quadriceps function in subjects with PFP. For instance, Bolgla et al. [7] and Rathleff et al. [9] reported increased amplitude of the EMG activity during stair descent in adults and adolescents with PFP as compared to asymptomatic subjects. On the other hand, decreased amplitude of the EMG activity was found by Møller et al. [17] and Toumi et al. [18] during knee extension efforts. These discrepant findings may be associated with the different nature of the tasks used in the aforementioned studies (e.g. knee extension efforts and stair negotiation). However, to date there is no study that has explored the amplitude of the quadriceps EMG activation in different activities performed by subjects with PFP. Such an investigation would clarify whether the neuromuscular system behaves differently as a function of the task and the presence of PFP.

Conflicting data have also been reported with regards to quadriceps strength. Several studies have found quadriceps weakness in subjects with PFP [8,11,19]. On the other hand, in the study of Toumi et al. [18] only 6 out of 32 subjects with PFP showed a significant deficit in quadriceps strength during maximal voluntary isometric contractions (MVIC), and Bolgla et al. [7] did not find quadriceps weakness in subjects with PFP during MVIC. Farina et al. [20] showed that experimental muscle pain caused decreased motor unit firing rate which were correlated to the pain intensity; and experimentally-induced knee pain (by injections of hypertonic saline) led to reductions in maximal knee extension and flexion strength that were positively correlated to pain intensity [21,22]. Therefore, a possible explanation for the controversial findings on quadriceps strength of individuals with PFP might be associated with the presence/absence of pain during the strength tests (i.e. due to variations in the amount of pain at the time of data collection), as intermittent symptoms are typically observed in subjects with PFP [23]. However, such a hypothesis is yet to be tested as no previous study has controlled the level of pain during assessments of quadriceps strength and neuromuscular function in individuals with PFP.

Therefore, the purposes of this study were: to (1) compare the amplitude of the EMG activity of the vastus medialis (VM) and vastus lateralis (VL) during MVIC and stair ascending tasks between women with and without PFP; (2) to compare the knee extensor strength during MVIC between women with and without PFP; and (3) investigate whether quadriceps function of women with PFP (as assessed by data of EMG and knee extension strength) behave differently before and after a loading protocol (i.e. stair walking) designed to aggravate symptoms.

## Methods

### Participants

Nineteen women with PFP and nineteen pain-free controls were recruited from public places of physical activity practice and gyms of the city. Only women were included due to the high prevalence of PFP in this population [4]. In addition, we assumed that including both sexes could be seen as a confounder factor as women are reported to exhibit different movement patterns than males [24]. Based on calculations made in Sample-power using Statistical Software for Social Sciences (SPSS) Version 18.0 (SPSS Inc. Chicago, IL, USA) with data from Sacco et al. [25], a minimum sample size of nineteen women per group was indicated to evaluate differences between groups for VM EMG amplitude (as measured by root mean square (RMS) values) with a statistical power of 80% and a significance level of 5%, observing a minimum difference of 13.78 mV between means with a standard deviation of 16.15 mV (effect size of *d* = 0.57; p = 0.0453). Prior to the data collection, all subjects provided written informed consent and the experimental protocol was approved by the Institutional Review Board of the São Paulo State University Human Ethics Committee (306.729).

Diagnosis of PFP was confirmed following consensus from two experienced clinicians (>5 years’ experience) and based on definitions used in previous studies [26–28]. The inclusion criteria were (1) anterior knee pain during at least 2 of the following activities: prolonged sitting, squatting, kneeling, running, climbing stairs, and jumping; (2) pain during patellar palpation; (3) symptoms of insidious onset and duration of at least 1 month; (4) worst pain level in the previous month at least 3cm on a 10cm visual analogue scale (VAS); and (5) 2 or more positive clinical signs in the following tests: Clarke’s sign, McConnell test, Waldron test and patellar pain on palpation. The subjects were required to fulfill all 5 requirements to be allocated to the PFP group. The subjects of the control group could not show any signs or symptoms of PFP or other musculoskeletal conditions. The presence of any of the following conditions were carefully screened as exclusion criteria: events of patellar subluxation or dislocation, lower limb inflammatory process, patellar tendon or meniscus tears, bursitis, ligament tears or the presence of neurological diseases. Those who had undergone knee surgery; or received oral steroids, opiate treatment, acupuncture or physiotherapy during the preceding 6 months were excluded from this study.

### Instrumentation

Subjects’ pain was assessed using a 10 cm VAS. This scale has been validated and it is considered a reliable tool for assessing pain in women with PFP [29]. The extreme left side of the VAS stated “no pain” whereas the extreme right side stated “worse pain imaginable.” Subjects drew a perpendicular line on the scale at the position that most likely described their pain.

The experimental design included a seven step staircase, each step being 28 cm deep and 18 cm high, with a walkway in front of and at the top of it. A force plate (AMTI, OR6, Watertown, MA, USA) was positioned in the center of the fourth step and used to obtain ground reaction force data and, thus, to establish the moment when the subject was passing over the step [26,30]. To ensure a natural stair ascent pattern, the subjects were not made aware of the force plate, which was hidden within the fourth step covered by a rubberized fabric, so that it was impossible to distinguish the force plate from the other steps [26,30]. The force plate acquisition sampling rate was 2000 Hz.

EMG data were collected using a conditioner module (Lynx, Sao Paulo, BRA; model 1000-8-4I) with a fourth-order, zero-lag, Butterworth digital filter (with cutoff frequencies set at 20 and 500 Hz) and an amplifier with a gain of 50. The preamplifier circuit on the electrode cable had a gain of 20, a common mode rejection ratio greater than 80 dB and an impedance of 1012 Ω. The raw EMG signal was recorded at a sampling rate of 4000 Hz [31]. Two pairs of bipolar surface-capture Ag/AgCl electrodes (Kendall, Mansfield, MA, USA; model Medi-Trace) with diameters of 10 mm were used to obtain VM and VL EMG data. The data were recorded using AqdAnalysis software (Lynx, Sao Paulo, SP, BRA; model EMG 1000-8-4I). An electrical stimulation device (Quark, Piracicaba, SP, BRA; model Nemesys 942) was used to find the VM and VL motor points.

All isometric strength testing was performed on a knee extensor chair (VITTALY, model convergent, São José do Rio Preto—SP, Brazil), a valid and reliable equipment for measuring strength and EMG data of the knee joint [32]. A uniaxial force transducer (model MM, KRATOS, Cotia—SP, Brazil) connected to a nonstretchable fabric strap was used to obtain knee extensor strength data. A specific channel of the module signal conditioner was set to acquire the signals from the force transducer. The electrical analog signal from the force transducer was amplified with a gain of 50, sampled at 1500 Hz using a 16-bit analog-to-digital converter and synchronized with the EMG signals. Force data of each MVIC trial was filtered using a fourth-order, zero-lag, Butterworth digital filter with a cutoff frequency of 10 Hz.

### Procedures

**Fig 1:**
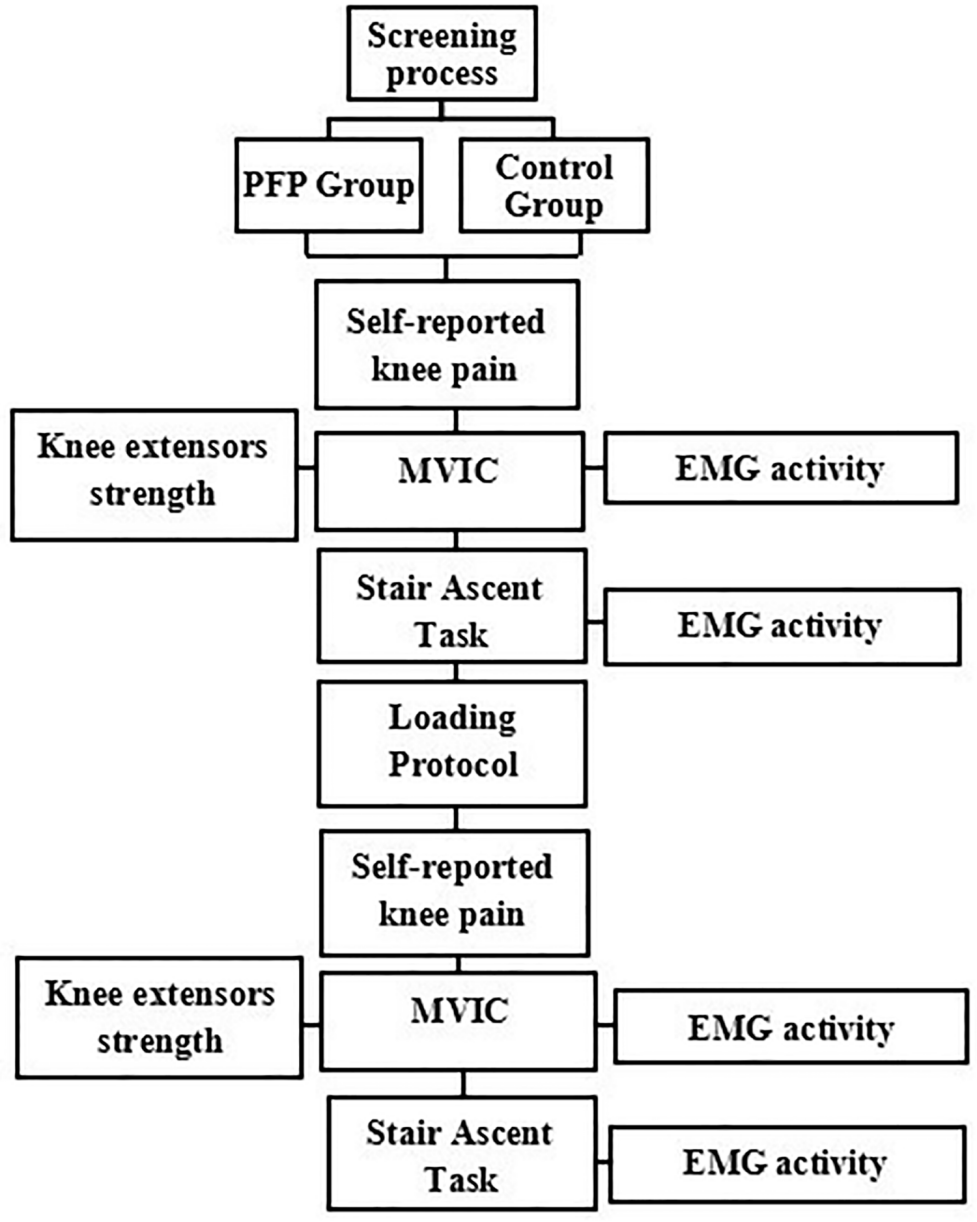
Flowchart exemplifying data collection. PFP = Patellofemoral pain; EMG = Eletromyographic; MVIC = Maximum Voluntary Isometric Contraction.

Fig 1 depicts a flowchart of the data collection. After finding the VM and VL motor points, the skin over the anterior portion of the thigh was cleaned with rubbing alcohol. The electrodes were placed 2 cm below the motor point in the direction of the muscle belly, with 20 mm between electrodes [10]. This motor point method for positioning the electrodes is in accordance with the Surface Electromyography for the Non-Invasive Assessment of Muscles (SENIAM) [33]. The reference electrode was placed over the tibial tubercle.

**Fig 2:**
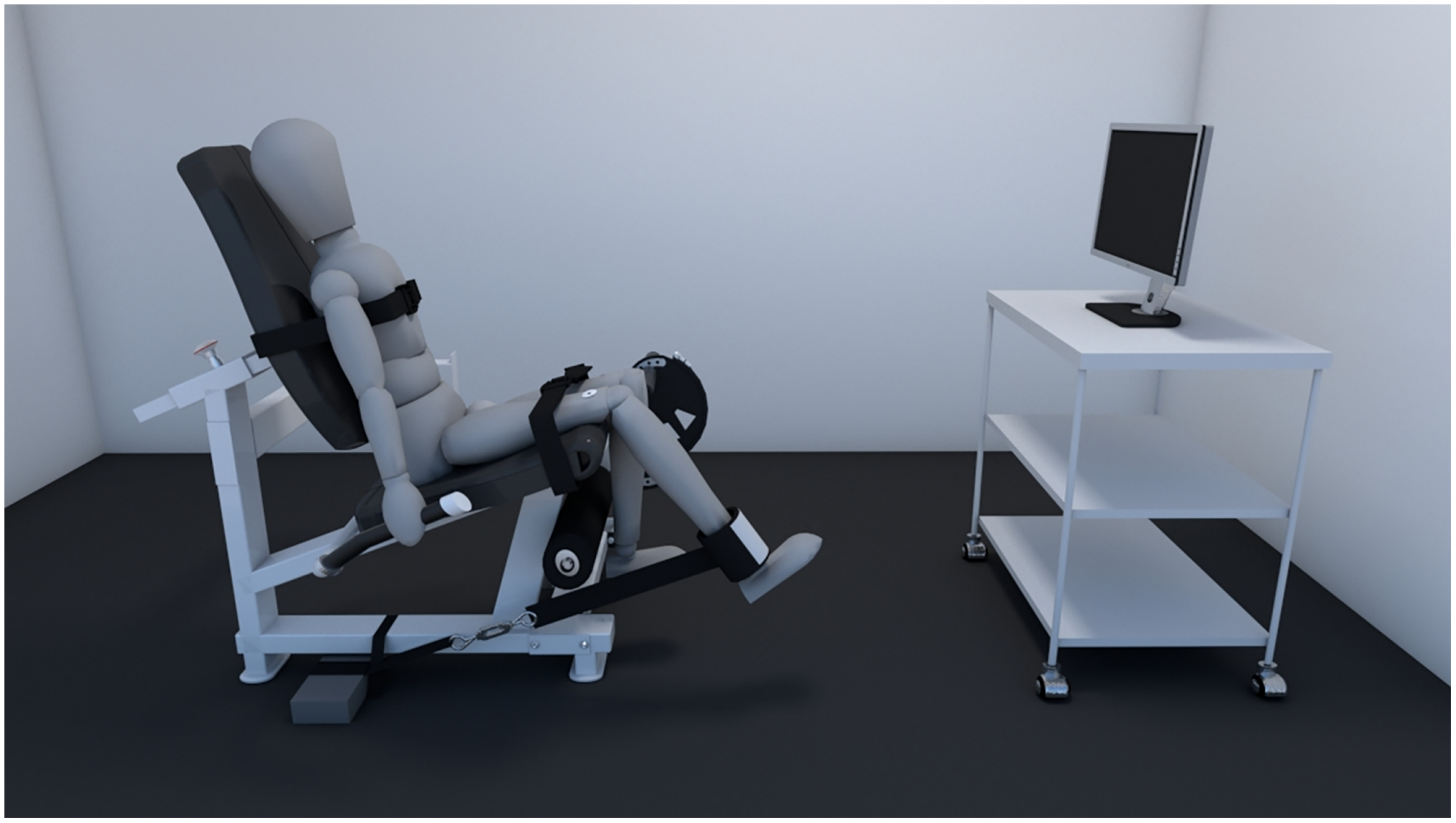
Schematic representation of the setup for the maximal voluntary isometric contraction (MVIC) assessments.

Prior to data collection, the subjects rated their level of pain on a VAS and were familiarized with the protocol. The subjects sat on the knee extensor chair with back adjustments and support (Fig 2). The knee-joint position was maintained at 60° and the hip was maintained at 90° of flexion in order to allow greater force generation of the quadriceps muscle [34,35]. Positions of the hip and the knee were checked using a universal goniometer. The trunk and knee were firmly strapped to the chair with a seatbelt. A testing strap was placed around the distal tibia 3 cm superior to the lateral malleolus as previously described [34,35]. The strap was then fastened to the force transducer, which was connected in series with a strap attached to a crossbar under the testing platform. A sliding plate connected to the crossbar allowed for positioning of the force transducer and fixation of the strap so that the line of pull of the transducer was perpendicular to the tibia. Before each trial, subjects were asked to apply a preload of 30 N to take slack out of the strap system. Three MVIC were performed for a six-second period and verbal encouragement was provided to induce the subjects to reach their maximal performance in each trial. Visual feedback of the produced force was provided. A two-minute rest was given between MVIC trials.

Then, subjects were asked to ascend the stair at their preferred speed to ensure a natural stair-climbing pattern and three successful trials were recorded. A trial was considered successful if the subject touched the force plate in the fourth step with her painful limb and with the entire foot. After the successful trials, participants were asked to climb and descend the stair 5 more times in order to exacerbate PFP symptoms (loading protocol) [36,37] and avoid any neuromuscular fatigue effect [5]. By the end of the loading protocol, the subjects rated their pain on the VAS and then performed the stair walking and MVIC tests again.

### Data processing

All processing was performed in MATLAB (The MathWorks, Inc, Natick, MA). The EMG signals of stair ascent were referenced by the vertical component of ground reaction force measured by the force plate, being a marker of the beginning and end of the EMG data collection. Therefore, the EMG signal was considered only while the participants were in contact with the fourth step [10]. The RMS of the EMG signal was then computed for the entire period over the fourth step (approximately 800-ms), for every 250-ms epochs (time window), and averaged among the three successful trials [38,39]. Then, RMS values were normalized to the EMG activity observed during the MVIC and presented as a percentage of the MVIC, as suggested in the literature [40]. For MVIC data, the first and last two seconds of the data were discarded from the analysis, either for EMG or for force transducer data (i.e. the middle two seconds remained for analysis) [35]. Muscle activity during MVIC was computed for every 250-ms epochs and averaged. This epoch (250-ms time window) was chosen in order to enhance EMG signal resolution and estimate more accurately EMG amplitude for both tasks [38,39], which is in line to what has been used in the literature [9]. In addition, the same epoch was used in the MVIC to avoid discrepancies in the method of analyzing the data.

The isometric force measurements (N) of the MVIC were recorded and were expressed as isometric torque (Nm). The distance from the lateral knee joint line (representative of the knee joint axis of rotation) to the lateral midpoint of the testing strap (approximately 35 cm) was measured for each subject and used as moment arm for the calculation of knee extensor torque. Knee joint line was determined by palpation with the knee slightly flexed and was considered as the joint space between Tibia and Femur along its lateral margin [41]. Although femoral condyles are known to be the axis of knee joint rotation, we have used the knee joint line for moment arm calculation given the high variability of femoral condyles curvature in humans [42], which increases error in identifying this landmark. The isometric torque was defined as the product of the isometric force and the moment arm. The peak torque produced during each trial was calculated and the average of the three trials was used for statistical analysis. This number of repetitions was chosen in order to avoid the influence of muscle fatigue on the data [43]. Torque data were normalized to body mass.

### Statistical analyses

The descriptive values (mean and SD) were obtained using SPSS. The data were analyzed as to its statistical distribution and variance homogeneity using the Shapiro–Wilk W test and Levene test, respectively. Independent t tests were used to investigate differences in the demographic data between groups. A 2-by-2 [group (with and without PFP) x time (before and after the loading protocol)] mixed model analysis of variance (ANOVA, with the factor “time” considered as repeated measures) was performed to investigate differences between and within groups for the EMG and torque during MVIC, EMG during stair walking and for the levels of pain (VAS scale). For all the ANOVA tests, significant main effects were reported if there were no significant interactions. If a significant interaction was found, the individual effects were analyzed separately. Bonferroni-adjusted t-tests were used to assess pairwise comparisons.

A Pearson product-moment correlation matrix was used to examine the relationships between the change in knee extensor torque and self-reported pain and between the change in EMG data during stair walking and self-reported pain. For this purpose, the score change from the first (before aggravating pain) to the second (after aggravating pain) MVIC and stair ascent evaluations was used in the statistics (i.e. second–first = Score change). Therefore, positive values indicate higher values in the second condition and negative values indicate higher values in the first condition. The guidelines for interpreting r values were according to Cohen [44]: 0.1 to 0.3 as small, 0.3 to 0.5 as moderate, and 0.5 to 1.0 as strong relations. The α > level was set at 0.05.

## Results

Independent t-tests for subject demographics revealed similar age, height, and body mass characteristics (Table 1).

**Table 1:** Anthropometric data of the subjects.

	All Mean (SD)	PFP Mean (SD)	CG Mean (SD)	t value	p-value
Age (y)	22.41 (2.97)	22.64 (2.62)	21.21 (3.12)	0.671	0.489
Height (m)	1.65 (0.08)	1.66 (0.07)	1.65 (0.05)	0.343	0.736
Weight (Kg)	60.38 (11.01)	60.02 (8.13)	61.39 (10.81)	0.956	0.198
N	38	19	19		

**Fig 3:**
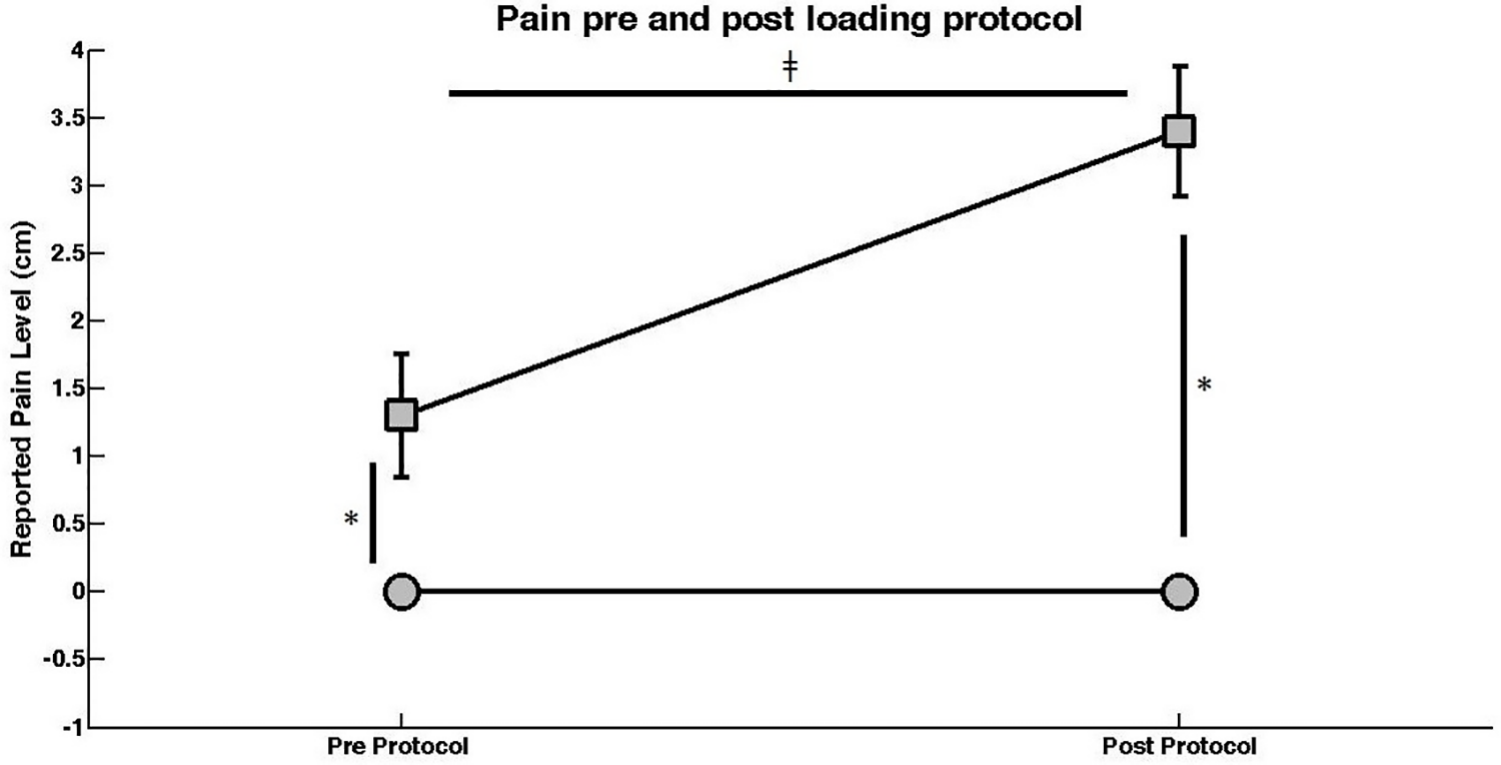
Self-reported pain before and after the loading protocol. * represents group differences. ǂ represents time differences. Circles = Control group. Squares = Patellofemoral pain group.

A significant group-by-time interaction was found among the times of pain evaluation and groups (F_(2,72)_ = 27.251, p = 0.000, η2 = 0.43) (Fig 3). The CG reported zero pain both before and after the loading protocol. On the other hand, the women of the PFP group reported an increase in the levels of pain from the first (1.3 ± 2.1) to the second (3.4 ± 2.0) pain measurement, indicating that the loading protocol (i.e. performance of stair walking) significantly increased their self-reported pain (which did not happen for the CG).

**Fig 4:**
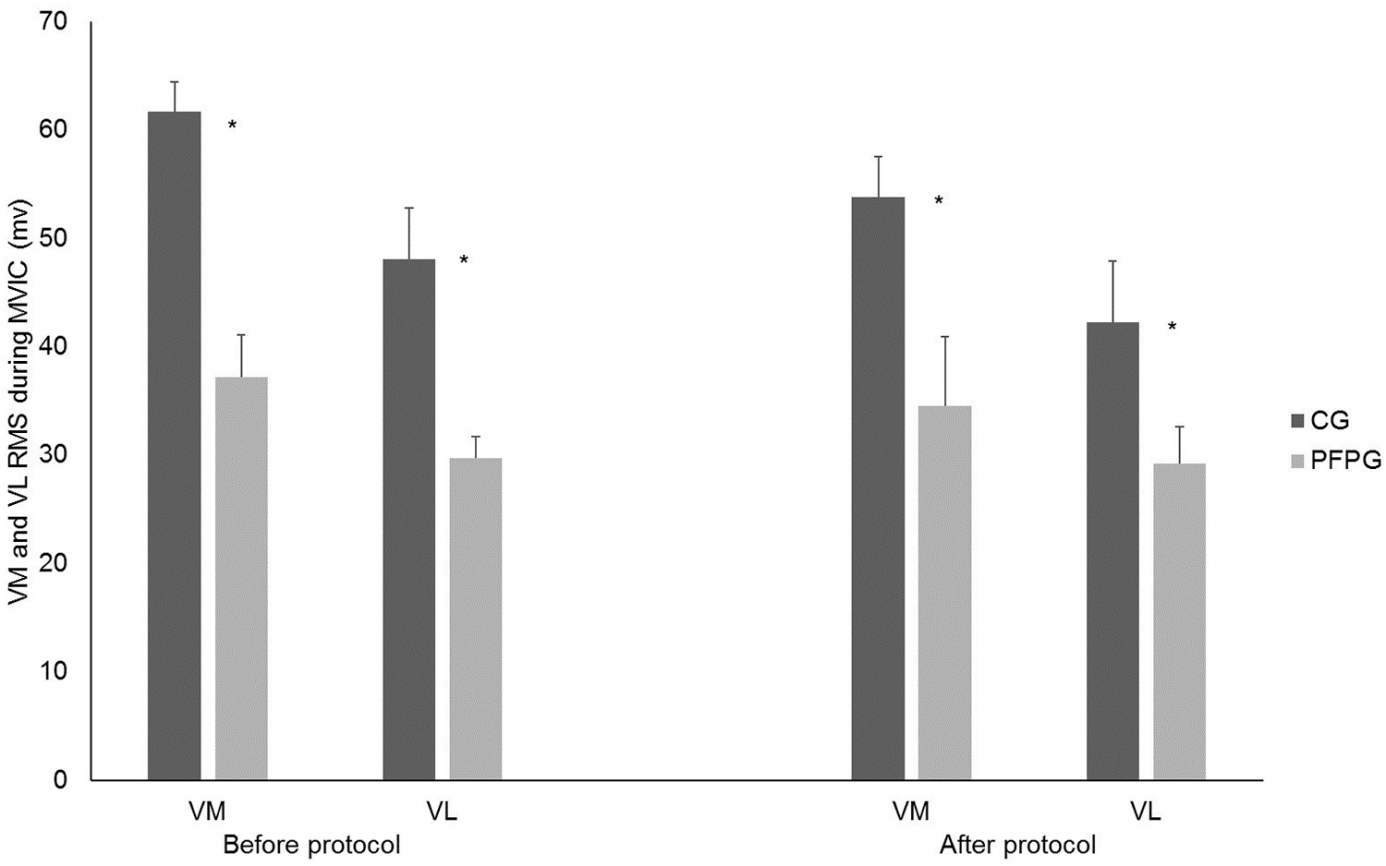
VM and VL EMG amplitude during MVIC performed before and after the loading protocol. VM = Vastus medialis; VL = Vastus Lateralis; EMG = Electromyographic; MVIC = Maximum voluntary isometric contraction; CG = Control group; PFP = Patellofemoral pain. * Represents group differences. No time differences were found.

The RMS values of VM and VL muscles during MVICs showed significant main effects of group (F_(1,36)_ = 10.869, p = 0.002, η2 = 0.23 and F_(1,36)_ = 4.985, p = 0.032, η2 = 0.12, for VM and VL, respectively) but not of time (F_(1,36)_ = 2.795, p = 0.103, η2 = 0.07 and F_(1,36)_ = 3.386, p = 0.074, η2 = 0.08, for VM and VL, respectively). In comparison with the CG, women with PFP showed lower RMS of VM and VL muscles in both MVIC tests (Fig 4).

**Fig 5:**
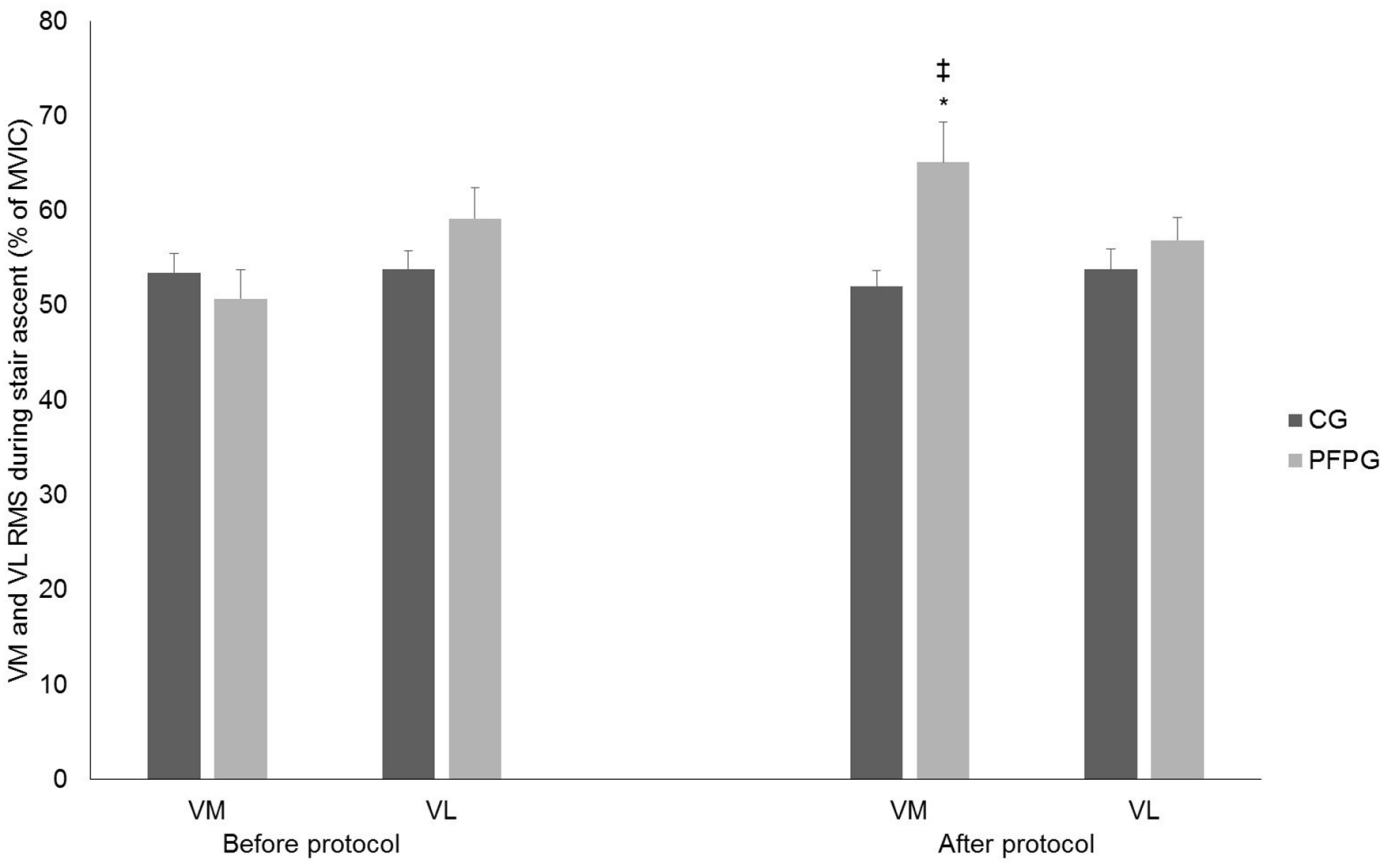
VM and VL EMG amplitude during the first (Pre Loading Protocol) and second (Post Loading Protocol) stair walking test. VM = Vastus medialis; VL = Vastus lateralis; EMG = Eletromyographic; CG = Control group; PFP = Patellofemoral pain. ‡ Represents time differences. * Represents group differences.

During the stair walking tests, there was a significant group-by-time interaction for the RMS values of VM EMG (F_(2,72)_ = 5.681, p = 0.023, η2 = 0.13) (Fig 5). During the first stair walking test (i.e. before the loading protocol), there was no difference between CG and PFP groups (p = 0.216). However, during the second stair walking test (i.e. after the loading protocol), there was a significant increase in the RMS values of VM EMG for the PFP group (p = 0.005) as compared to the same parameter before the loading protocol. This was accompanied by a significant difference in the RMS values of VM EMG between groups (p = 0.007), i.e. during the stair walking test performed after the loading protocol, RMS values of VM EMG were larger in the PFP group as compared to the CG. On the other hand, neither interaction nor main effects of group and time were found in the RMS values of VL EMG.

**Fig 6:**
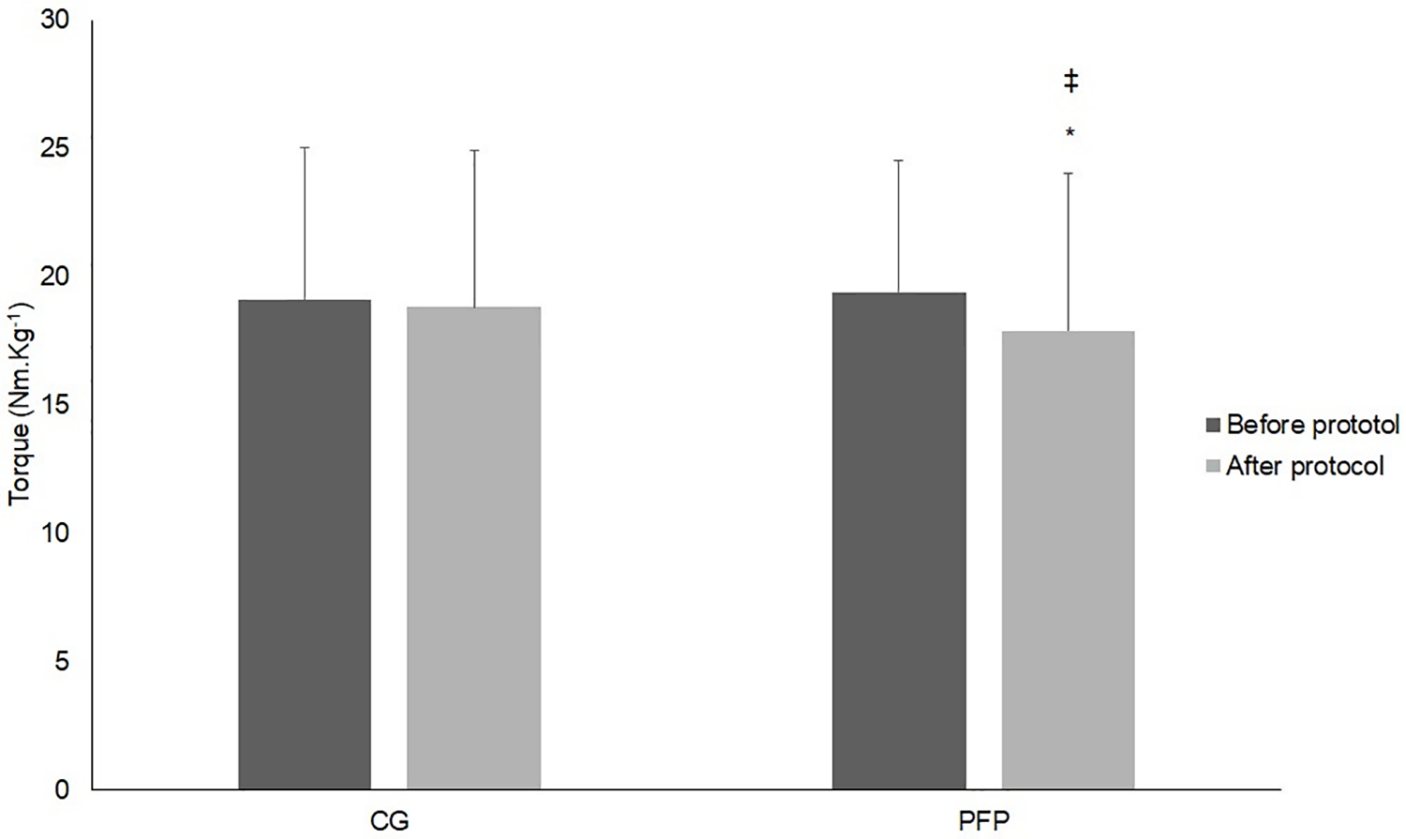
Knee extensors strength during MVIC performed before and after the loading protocol. MVIC = Maximum voluntary isometric contraction. * Represents group differences. ǂ Represents time differences.

A significant group-by-time interaction was observed for knee extensor torque (F_(2,72)_ = 7.713, p = 0.049, η2 = 0.10) (Fig 6). On the first MVIC test there was no difference between groups (p = 0.582) (PFP = 19.4 ± 6.1; CG = 19.1 ± 5.9). However, on the second MVIC test, women with PFP presented significantly lower maximum isometric torque of the knee extensors as compared to the CG (p = 0.002) (PFP = 17.9 ± 6.1; CG = 18.9 ± 5.1), which was also lower in the PFP group after the loading protocol as compared to before the loading protocol (p = 0.032).

**Fig 7:**
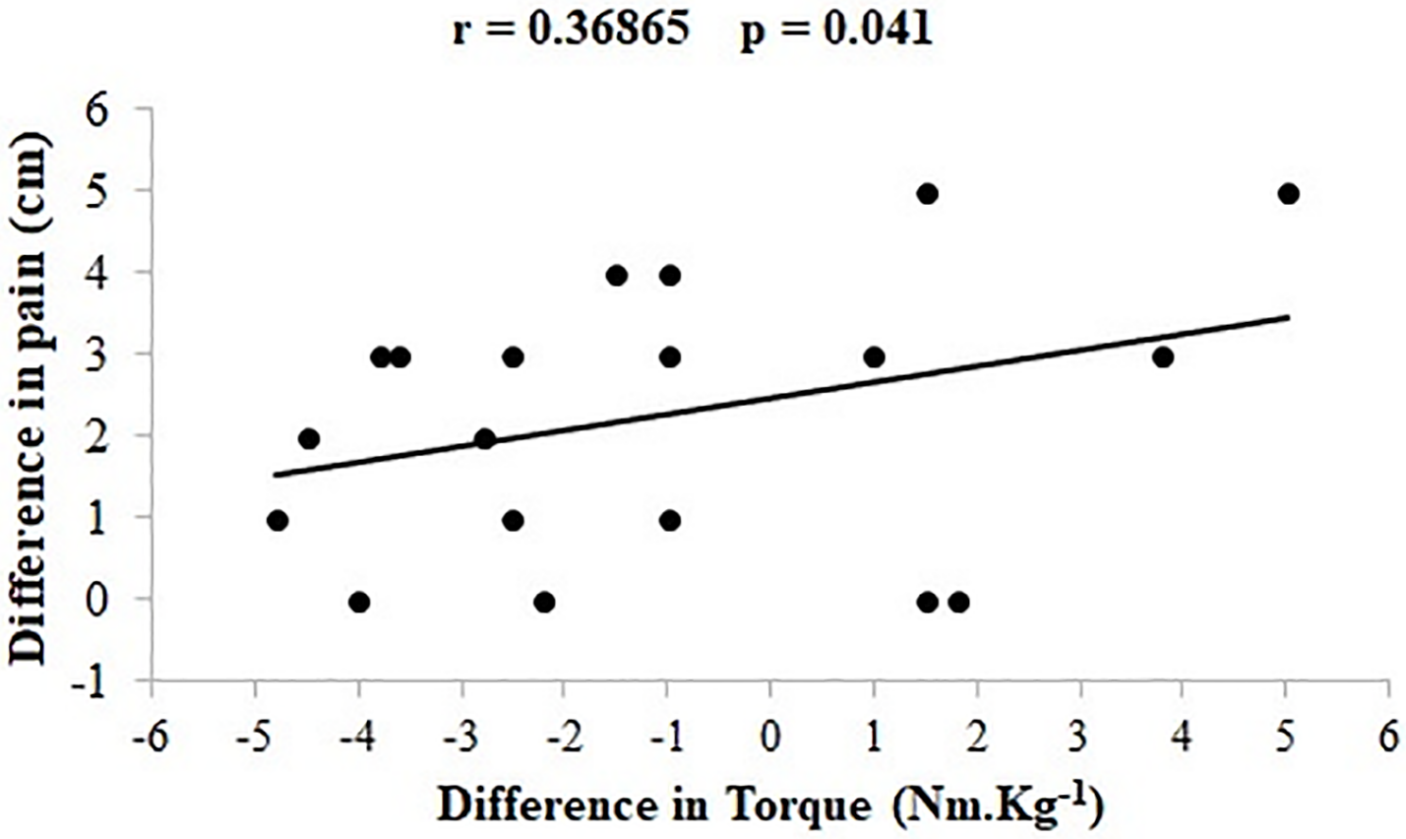
Correlation between changes in the knee extensors strength (MVIC) and changes in self reported pain from the assessment made before the loading protocol to the one made after the loading protocol. MVIC = Maximum voluntary isometric contraction. Negative and positive values indicate lower and higher knee extensor strength and self reported pain after the loading protocol, respectively.

**Fig 8:**
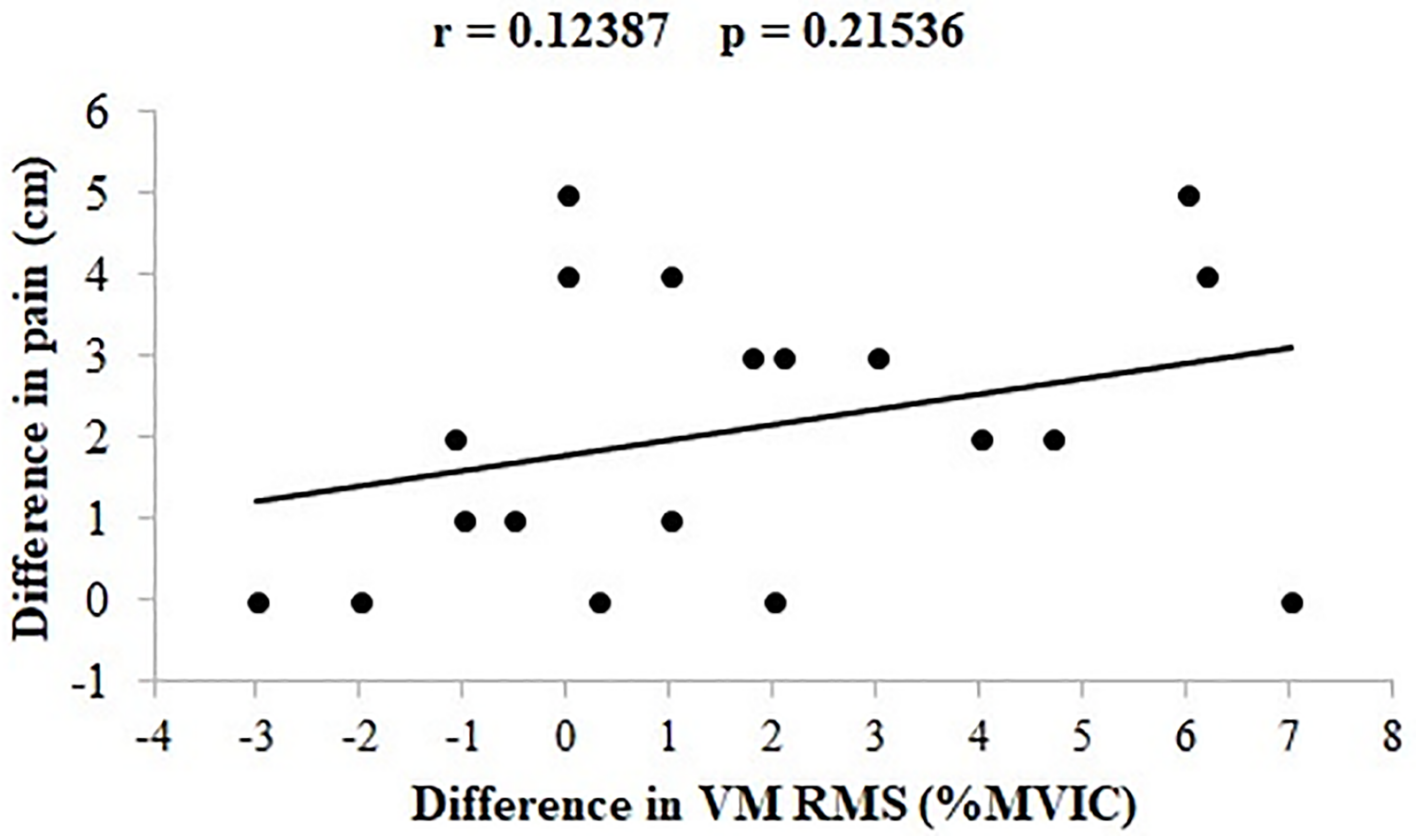
Correlation between the change of the VM RMS and pain from the first to the second stair walking test. VM = Vastus medialis, RMS = Root mean square. Negative and positive values indicate lower and higher VM RMS and self reported pain after the loading protocol, respectively.

Lastly, there was a significant moderate correlation between the changes of self-reported pain levels and knee extensor torques from the MVIC tests performed by the PFP group before and after the loading protocol (p = 0.041, r = 0.37) (Fig 7). No significant correlation was found between the changes in the self-reported pain levels and RMS values of VM from stair walking tests performed by the PFP group before and after the loading protocol (p = 0.215, r = 0.12) (Fig 8).

## Discussion

The purpose of this study was to determine whether women with PFP have altered amplitude of the EMG activity of the VM and VL during MVIC and stair ascending as compared to controls. In addition, we aimed to determine whether women with PFP present lower knee extensor strength during MVIC than healthy controls. Lastly, this study investigated whether quadriceps function of women with PFP (as assessed by data of EMG and knee extension strength) behave differently before and after a loading protocol (i.e. stair walking) designed to aggravate symptoms. In comparison to controls, women with PFP displayed lower levels of VM and VL EMG activation during MVIC assessments, irrespective to the moment of evaluation (i.e. before or after the loading protocol). On the other hand, higher levels of VM muscle activity were found during a functional stair climbing task, which, however, happened exclusively during the assessments performed after the loading protocol. Similarly, women with PFP displayed lower knee extensor strength only during the MVIC tests performed after the loading protocol (i.e. no differences in knee extensor strength were observed between PFP and CG before the loading protocol). Interestingly, the reductions in knee extensor strength from the assessments performed before to after the loading protocol were significantly associated with the increases in the levels of self-reported pain, while the changes in EMG activity were not associated with the increase in pain reports.

The results of the present study may elucidate some controversial findings of the literature. It really seems that the type of task investigated in the studies’ design may play a role on the quadriceps activation of women with PFP. Our results from EMG activity during MVIC corroborated with previous studies [17,18], showing lower amplitude of VM and VL EMG activity in women with PFP as compared to controls. Møller et al.[17] found a 20% decrease in the VM and VL EMG activity during MVIC as compared to nonsymptomatic knees, which is in line with our findings of 30% of decrease. These findings may be interpreted in light of recent studies that reported lower excitability of the monosynaptic reflex pathway of the VM muscle in women with PFP as compared to controls [45], which is related with pain, function and chronicity [46]. As shown by Park and Hopkins [47], both voluntary and involuntary inhibitory pathways may be involved in such a reduced motoneuronal excitability, and hence women with PFP may not be able to recruit the entire pool of motoneurons during maximum quadriceps efforts, which may be related to inhibitory neural mechanisms [45,46].

On the other hand, VM EMG activity in the PFP group increased in the stair walking test performed after the loading protocol, which did not happen for the VL muscle. VM EMG activity increased from 50% to 70% of the MVIC after the loading protocol. This is in line with Bolgla et al. [7] who found that individuals with PFP had 80% of VM EMG activity (normalized by the MVIC) during stair stepping, although in their study the participants did not perform any previous activity of the test as done in the present study. However, the increase in VM EMG activation during the stair ascent task performed after the loading protocol did not correlate with the increase in pain. Denning et al. [48] have found that experimentally-induced knee pain during running actually decreased VM and VL EMG amplitude. As such, pain itself might not be responsible for the increase in VM EMG amplitude found in the present study. Rather, the increase in VM EMG activation may reflect a compensatory strategy to complete the stair ascent task [9], an attempt to medialize the patella and decrease the PFJ stress [9,10], or even a compensatory strategy to recruit a weakened muscle [7]. Therefore, it seems that neurophysiological inhibitory mechanisms aforementioned may lead to lowered muscle activation only during activities that demand maximum quadriceps efforts, whereas during functional activities such as stair ascent women with PFP may be able to increase quadriceps activation as part of a compensatory/protective strategy [9]. These results are in line with previous investigation in which mild and moderate experimental pain differently affected EMG and kinematic profiles of elbow-flexion movements, especially for high-force demand tasks [49]; and also with previous studies with experimental pain that provided the notion that central inhibitory motor control mechanisms act as a function of the intensity of the nociceptive activity [20,22]. Given as such, the neuromuscular system seems to behave differently as a function of the task and the presence of PFP.

The interaction between trials of stair ascent shows the dosing effect to elicit elevated EMG amplitude, which corroborates with previous findings showing that PFP and its compensatory mechanisms are typically exacerbated by activities requiring loading on a flexed knee, such as stair ascent [5,37]. Given as such, performing biomechanical and clinical analyzes after a set of activities that exacerbate PFP symptoms would probably provide different outcomes than without doing it [5,10]. These differences could be a confounder factor for clinicians as patients can attend a clinical appointment under a variety of conditions, including after the performance of knee loading activities (e.g. running) or after a day of rest. Our findings demonstrate that patients inserted in such scenario would present different outcomes depending on the activity performed before evaluation.

As compared to controls, women with PFP did not show quadriceps weakness during the first MVIC test (performed before the loading protocol). However, after the loading protocol, women with PFP demonstrated significantly less knee extensor strength. Such a decreased strength was accompained by an increase on self-reported pain, with a significant moderate correlation between the changes in knee extensor strength and self-reported pain from the first (before the loading protocol) to the second MVIC test (after the loading protocol). These results corroborate with Powers et al. [8] who found that individuals with PFP performed lower knee extensor torque of 23.6 N.cm/Kg (confidence interval = 12.8–39.2) compared to controls who did 30.4 N.cm/Kg (confidence interval = 19.6–40.2). As proposed by Bolgla et al. [7], quadriceps weakness may be more prominent in women with PFP when they are experiencing pain. This hypothesis is supported by the findings of the present study and those from Park and Hopkins’ [47], who showed that experimentally induced anterior knee pain (ie, with hypertonic saline injection in asymptomatic subjects) caused a 12% decrease in maximal isometric knee extension torque, which is also in line with the results of Henriksen et al. [22], in wich experimental knee pain generated reductions in maximal knee strength that were correlated with pain intensity. Therefore, care should be taken in evaluating quadriceps strength in women with PFP as different outcomes may be observed as a function of the pain level, especially considering that intermittent symptoms are typically observed in subjects with PFP [23].

This study has some limitations that should be acknowledged. There are other factors that might have played a role in the discrepancies between studies’ results which could not be considered in the present study, such as physical activity level, demographic characteristics of the sample, pain-related anxiety and data analysis techniques. In addition, the sample of this study included only women with PFP, which may limit the generalizability of the results to men and adolescents.

## Conclusion

This study found that women with PFP display lower levels of quadriceps EMG activation during maximal knee efforts, but higher EMG activation during dynamic tasks (stair ascent) after pain exacerbation. Additionally, women with PFP display lower knee extensor strength only after pain exacerbation, which was moderately associated with the increase in self-reported pain, whereas the changes in EMG activity during stair ascent were not correlated with self-reported pain. Thus, quadriceps weakness in women with PFP may be exacerbated when they are experiencing knee pain and hence care should be taken by clinicians during quadriceps strength evaluation in women with PFP, as misleading outcomes may appear if the intensity of knee pain is not considered during screening. These results also suggest that rehabilitation programs should focus on both restoring neuromuscular control and increasing muscle strength.
