# Correction: Quadriceps neuromuscular function in women with patellofemoral pain: Influences of the type of the task and the level of pain

**DOI:** 10.1371/journal.pone.0207421

**Published:** 2018-11-08

**Authors:** Ronaldo Valdir Briani, Danilo De Oliveira Silva, Carolina Silva Flóride, Fernando Amâncio Aragão, Carlos Eduardo de Albuquerque, Fernando Henrique Magalhães, Fábio Mícolis de Azevedo

There are errors in the Funding section. The correct funding information is as follows: This work was supported by the São Paulo Research Foundation (FAPESP) with grants to FMA (2014/24939-7) and FHM (2015/13096-1), and a scholarship to RVB (2015/00406-2). The funder had no role in study design, data collection and analysis, decision to publish, or preparation of the manuscript.
